# Consensus for management of sacral fractures: from the diagnosis to the treatment, with a focus on the role of decompression in sacral fractures

**DOI:** 10.1186/s10195-023-00726-2

**Published:** 2023-09-04

**Authors:** Alessandro Aprato, Luigi Branca Vergano, Alessandro Casiraghi, Francesco Liuzza, Umberto Mezzadri, Alberto Balagna, Lorenzo Prandoni, Mohamed Rohayem, Lorenzo Sacchi, Amarildo Smakaj, Mario Arduini, Alessandro Are, Concetto Battiato, Marco Berlusconi, Federico Bove, Stefano Cattaneo, Matteo Cavanna, Federico Chiodini, Matteo Commessatti, Francesco Addevico, Rocco Erasmo, Alberto Ferreli, Claudio Galante, Pietro Domenico Giorgi, Federico Lamponi, Alessandro Moghnie, Michel Oransky, Antonio Panella, Raffaele Pascarella, Federico Santolini, Giuseppe Rosario Schiro, Marco Stella, Kristijan Zoccola, Alessandro Massé

**Affiliations:** 1 Università degli studi di Torino, Viale 25 Aprile 137 Int 6, 10133 Turin, Italy; 2 APSS Trento, Trento, Italy; 3 Università degli studi di Brescia, Brescia, Italy; 4 Università Cattolica del Sacro Cuore, Rome, Italy; 5 ASST Grande Ospedale Metropolitano Niguarda di Milano, Milan, Italy; 6 Università degli studi di Milano, Milan, Italy; 7 Tanta University, Tanta, Egypt; 8 Ospedale Sant’Eugenio di Roma, Rome, Italy; 9 Policlinico Casilino di Roma, Rome, Italy; 10 Ospedale Mazzoni di Ascoli, Ascoli Piceno, Italy; 11 Humanitas Research Hospital Rozzano, Rozzano, Italy; 12 ASST degli spedali Civili di Brescia, Brescia, Italy; 13 ASST Papa Giovanni XXIII di Bergamo, Bergamo, Italy; 14 Ospedale Maggiore di Bologna, Bologna, Italy; 15 Ospedale Civile Santo Spirito di Pescara, Pescara, Italy; 16 Università degli studi di Cagliari, Cagliari, Italy; 17 Ospedale Augusto Murri di Fermo, Fermo, Italy; 18 Università degli studi di Bologna, Bologna, Italy; 19 Università degli studi di Roma, ASST degli spedali Civili di Brescia, Brescia, Italy; 20 Università degli studi di Bari, Bari, Italy; 21 Ospedali Riuniti di Ancona/Università degli studi di Ancona, Ancona, Italy; 22 Università degli studi di Genova, Genoa, Italy; 23 Ospedale SS Antonio e Biagio di Alessandria, Alessandria, Italy

**Keywords:** Sacral fracture, Decompression, Laminectomy, Cauda equina syndrome, Pelvic injuries

## Abstract

**Background:**

There is no evidence in the current literature about the best treatment option in sacral fracture with or without neurological impairment.

**Materials and methods:**

The Italian Pelvic Trauma Association (A.I.P.) decided to organize a consensus to define the best treatment for traumatic and insufficiency fractures according to neurological impairment.

**Results:**

Consensus has been reached for the following statements: When complete neurological examination cannot be performed, pelvic X-rays, CT scan, hip and pelvis MRI, lumbosacral MRI, and lower extremities evoked potentials are useful. Lower extremities EMG should not be used in an acute setting; a patient with cauda equina syndrome associated with a sacral fracture represents an absolute indication for sacral reduction and the correct timing for reduction is “as early as possible”. An isolated and incomplete radicular neurological deficit of the lower limbs does not represent an indication for laminectomy after reduction in the case of a displaced sacral fracture in a high-energy trauma, while a worsening and progressive radicular neurological deficit represents an indication. In the case of a displaced sacral fracture and neurological deficit with imaging showing no evidence of nerve root compression, a laminectomy after reduction is not indicated. In a patient who was not initially investigated from a neurological point of view, if a clinical investigation conducted after 72 h identifies a neurological deficit in the presence of a displaced sacral fracture with nerve compression on MRI, a laminectomy after reduction may be indicated. In the case of an indication to perform a sacral decompression, a first attempt with closed reduction through external manoeuvres is not mandatory. Transcondylar traction does not represent a valid method for performing a closed decompression. Following a sacral decompression, a sacral fixation (e.g. sacroiliac screw, triangular osteosynthesis, lumbopelvic fixation) should be performed. An isolated and complete radicular neurological deficit of the lower limbs represents an indication for laminectomy after reduction in the case of a displaced sacral fracture in a low-energy trauma associated with imaging suggestive of root compression. An isolated and incomplete radicular neurological deficit of the lower limbs does not represent an absolute indication. A worsening and progressive radicular neurological deficit of the lower limbs represents an indication for laminectomy after reduction in the case of a displaced sacral fracture in a low-energy trauma associated with imaging suggestive of root compression. In the case of a displaced sacral fracture and neurological deficit in a low-energy trauma, sacral decompression followed by surgical fixation is indicated.

**Conclusions:**

This consensus collects expert opinion about this topic and may guide the surgeon in choosing the best treatment for these patients.

*Level of Evidence*: IV.

*Trial registration*: not applicable (consensus paper).

## Background

Sacral fractures are frequently associated with concomitant neurological injury and range from incomplete radiculopathies to a complete cauda equina syndrome depending on the mechanism of trauma, fracture type and location [1, 16, 20, 21, 43, 49, 53, 54], considering that they can reach up to 62% neurological impairment in sacral transverse fractures [24]. Sacral fractures are estimated to occur in 45% of all pelvic fractures; 4.5% are transverse. Less than 5% of sacral fractures occur as isolated injuries, often resulting from a direct blow or fall onto the sacrum. Because of the location of the lumbosacral plexus with respect to the sacrum, 25% of sacral fractures are associated with a neurologic injury. The widely accepted classification system of vertical sacral fractures was proposed by Denis and based on the location of the fracture (lateral to, through or medial to neural foramina; zones I–III) and their association with neurologic injury [2, 20]. Medialization of the fracture line and presence of additional transverse sacral fractures increase the prevalence of concomitant neurologic injury [3, 25, 62, 65]. Subsequent sacral classifications are mainly concerned with fracture morphology and do not consider neurologic injury as a determinant affecting surgical management [4, 30, 31]. Recently, efforts have begun to develop a comprehensive sacral fracture classification system that integrates neurologic status as a major determinant of the indication for surgical intervention [5, 17, 22, 26, 42, 47, 64]. Present guidelines for traumatic neurologic injury in patients with ongoing neural element compression have been shaped basically by the Surgical Timing in Acute Spinal Cord Injury Study (STASCIS) trial [6]. Therefore, there is great interest in having an orthopaedic point of view on this topic. The sacrum has a unique biomechanical feature because of the lack of segmental motion. Moreover, many sacral fractures are treated by trauma surgeons without formal spine surgery training [8]. At the moment there is no consensus on neurological monitoring, timing or type of surgical intervention (direct or indirect compression, with or without fixation) in complete and incomplete sacral neurological injuries [14, 15, 58–61].

A consideration that is worth subscribing to is that sacral fractures can occur in high-energy trauma in association with some other lesions of the pelvis, rachis or other districts; but, still, they can also occur in low-energy trauma in old patients, the so-called insufficiency fractures. Sacral insufficiency fractures can also result in neurological impairment, but little is known from the present literature about this kind of setting. Some authors suggest a possible role of sacral decompression in progressive neurological deficit associated with sacral insufficiency fractures, but with a lack of data supporting this strategy, the debate continues [8, 55, 56].

## Methods

Regulations used in order to conduct the Consensus Conference (CC) were adopted from “*The Methodological Manual — How to Organise a Consensus Conference*”. Levels of evidence (LoE) come from the Oxford Centre for Evidence-based Medicine.

The organizing committee undertook the critical revision of the literature: five authors independently performed a Higher Health Institute systematic literature review according to PRISMA statements. Medical Subject Headings (MeSH) terms were used with the search strings: “Sacral” and “fractures” and “decompression” or “neurological deficit” or “timing”; “sacral fractures” and “neurological decompression”; “sacral fractures” “surgery”; “insufficiency sacral fractures” and “treatment”.

These terms were sequentially searched using the following databases: MEDLINE, PubMed, EMBASE, Scopus, and Cochrane Database of Systematic Reviews. Databases are the main tools for researching the literature. The following inclusion criteria were used: all articles focusing on the management of sacral fractures associated with or not associated with a neurological deficit were considered from case report to meta-analysis, considering the level of statistical relevance and populations of the various studies.

Inclusion criteria consisted of published studies pertinent to our research question between the years 1977 and 2023. Results were limited to humans and to papers published in the English language, although some studies in French were also included.

Conferences, abstracts, theses, unpublished reports and commercial advertisements were excluded as the level of evidence was considered too low.

**Fig. 1:**
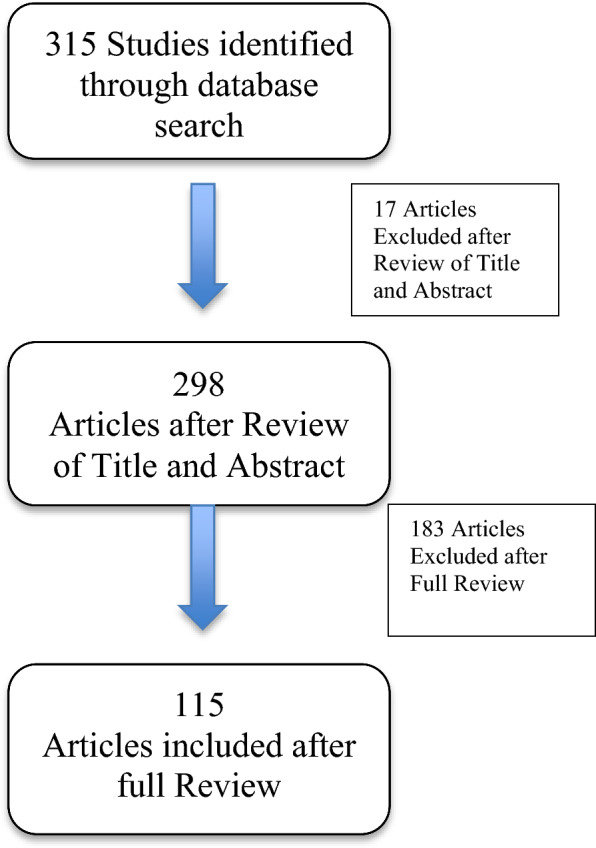
Diagram showing selection of the articles based on inclusion and exclusion criteria

Initially, titles of articles which met the inclusion criteria were screened for primary inclusion. All the obtained abstracts were further evaluated for acceptability. The full texts of articles which met the relevance and inclusion criteria were obtained and reviewed, paying particular attention to relevance to our research questions. A manual cross reference search of relevant studies was performed, and the related relevant papers were also retrieved. The acquisition of articles is summarised in the flow-chart diagram (Fig. 1).

After the literature searches, the authors provided a comprehensive summary document divided into 20 un-solved questions summarized in 6 sections: diagnostic process, sacral fracture and cauda equina syndrome, sacral fracture and peripheral nerve deficit, late decompression, surgical technique and insufficiency fractures. Those 20 questions were discussed with the society board panel and the following 20 recommendations were proposed.

## Proposed recommendations

Section 1: Diagnostic process

1. If a complete neurological examination (e.g. intubated polytraumatized patient) cannot be performed, pelvic X-rays are useful.
2. If a complete neurological examination (e.g. intubated polytraumatized patient) cannot be performed, a CT scan is useful.
3. If a complete neurological examination (e.g. intubated polytraumatized patient) cannot be performed, hip and pelvis MRI is useful.
4. If a complete neurological examination (e.g. intubated polytraumatized patient) cannot be performed, lumbosacral MRI is useful.
5. If a complete neurological examination (e.g. intubated polytraumatized patient) cannot be performed, lumbosacral evoked lower extremities potentials are useful.
6. Lower extremities EMG should not be used in an acute setting.

Section 2: Sacral fracture and cauda equina syndrome

7. A patient with cauda equina syndrome (lower extremities neurological deficit, erectile dysfunction, urinary retention/urinary or faecal incontinence and saddle anaesthesia) associated with a sacral fracture represents an absolute indication for sacral reduction and fixation.
8. In a patient with cauda equina syndrome (lower extremities neurological deficit, erectile dysfunction, urinary retention/urinary or faecal incontinence and saddle anaesthesia) associated with a sacral fracture, the correct timing for reduction and fixation is “as early as possible”.

Section 3: Sacral fracture and peripheral nerve deficit

9. An isolated and complete radicular neurological deficit of the lower limbs represents an indication for laminectomy after reduction in the case of a displaced sacral fracture in a high-energy trauma associated with imaging suggestive of root compression.
10. An isolated and incomplete radicular neurological deficit of the lower limbs does not represent an indication to laminectomy after reduction in the case of a displaced sacral fracture in a high-energy trauma associated with imaging suggestive of root compression.
11. Worsening and progressive radicular neurological deficit of the lower limbs represents an indication for laminectomy after reduction in the case of a displaced sacral fracture in a high-energy trauma associated with imaging suggestive of root compression.
12. In the case of a displaced sacral fracture and neurological deficit with imaging showing no evidence of nerve root compression, a laminectomy after reduction is not indicated.

Section 4: Late decompression

13. In a patient who was not initially investigated from a neurological point of view (neither physical examination nor imaging), due to general circumstances, but then clinical investigation after 72 h identifies a neurological deficit in the presence of a displaced sacral fracture with nerve compression on MRI, a laminectomy after reduction is indicated.

Section 5: Surgical technique

14. If there is an indication to perform a sacral decompression, a first attempt with closed reduction through external manoeuvres is not mandatory.
15. Transcondylar traction does not represent a valid method of closed decompression in unstable unilateral pelvic fractures and vertical-shear type fractures.
16. Following a sacral decompression, a sacral fixation (e.g. sacroiliac screw, triangular osteosynthesis, lumbopelvic fixation) should be performed.

Section 6: Insufficiency fractures

17. An isolated and complete radicular neurological deficit of the lower limbs represents an absolute indication for laminectomy after reduction in the case of a displaced sacral fracture in a low-energy trauma associated with imaging suggestive of root compression.
18. An isolated and incomplete radicular neurological deficit of the lower limbs does not represent an absolute indication for laminectomy after reduction in the case of a displaced sacral fracture in a low-energy trauma associated with imaging suggestive of root compression.
19. Worsening and progressive radicular neurological deficit of the lower limbs represents an indication for laminectomy after reduction in the case of a displaced sacral fracture in a low-energy trauma associated with imaging suggestive of root compression.
20. In the case of a displaced sacral fracture and neurological deficit in a low-energy trauma, surgical fixation after performing a sacral decompression is indicated.

All the active members of the society were interviewed as to their agreement or disagreement with the proposed statements. Consensus was reached for each statement if the level of agreement among the members was over 75%. Subsequently, the results were discussed during the annual meeting of the Italian Pelvic Trauma Association.

## Results

**Fig. 2:**
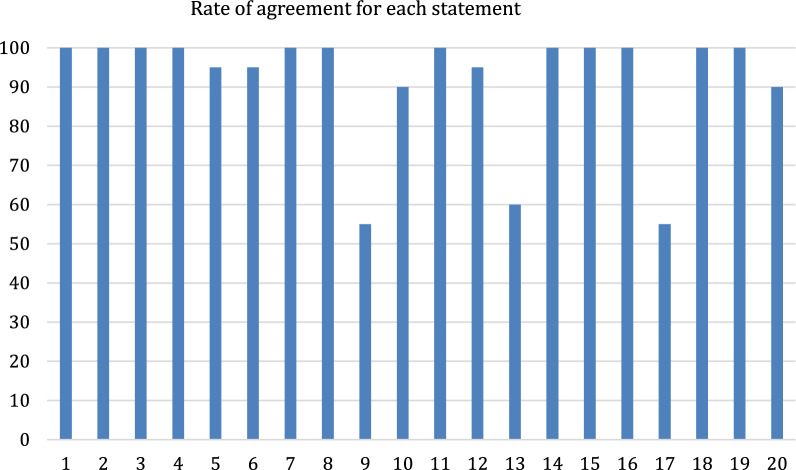
Rate of agreement for each statement

Agreement rates for each statement are shown in Fig. 2 and the statements approved by consensus are summarized in Table 1. The panel did not reach consensus about the following statements:

- An isolated and complete radicular neurological deficit of the lower limbs represents an indication for laminectomy after reduction in the case of a displaced sacral fracture in a high-energy trauma associated with imaging suggestive of root compression.
- In a patient who was not initially investigated from a neurological point of view (neither physical examination nor imaging), due to general circumstances, but then clinical investigation after 72 h identifies a neurological deficit in the presence of a displaced sacral fracture with nerve compression on MRI, a laminectomy after reduction is indicated.
- An isolated and complete radicular neurological deficit of the lower limbs represents an absolute indication for laminectomy after reduction in the case of a displaced sacral fracture in a low-energy trauma associated with imaging suggestive of root compression.

In the following paragraph, comments by the panel during the final discussion are reported.

The panel emphasized that lumbopelvic CT scan with multiplanar reconstruction is considered strictly necessary for a correct diagnosis and surgical planning. Furthermore, the panel also agreed that hip, pelvis and lumbosacral MRI (Magnetic Resonance Imaging) is useful for detecting radicle kinking and compression of the nerve/plexus by the displaced fracture, and it should be performed as early as possible considering the general condition of the patient. Eventually, the panel agreed that timing for all examinations should be chosen according to the patient’s general condition.

Regarding statement 6, the panel suggested that EMG may have a clinical application only after at least 3–4 weeks in patients with early deficit, to monitor eventual neurological recovery.

Even if there is no actual evidence that a sacral decompression performed less than 72 h from trauma achieves a better outcome [2], the panel suggested that reduction should be conducted as early as possible, considering the general condition of the patient and type of fracture [46, 47].

Experts agreed in considering an open surgical decompression (reduction and subsequent possible laminectomy ± fixation) useful where, on the basis of the pre-operative images, a closed decompression would not be conceivable.

In the case of a sacral decompression, a closed reduction for external manoeuvres should be considered only in selected cases; furthermore, external manoeuvres may be indicated only if the patient’s general conditions are good enough to allow this treatment in the first 48 h [14, 15].

A consensus was reached regarding the necessity of performing a stabilization after a sacral decompression, but not regarding which type of stabilization technique was the best; the debate is still going on, mainly in comparing sacral screw vs triangular fixation. The panel did not reach consensus regarding the scenario of an isolated and complete radicular neurological deficit of the lower limbs.

## Conclusions

According to the consensus, the best way to approach a patient with a suspected neurological impairment associated with a displaced sacral fracture should be based on at least an AP X-ray of the pelvis, eventually followed by CT, useful especially for surgery planning. Pelvic and lumbosacral MRI (Magnetic Resonance Imaging) as well as evoked lower extremities can be useful tools for completing the diagnostic approach if a patient’s general conditions allow [7, 10].

Several articles have shown that radiological investigations including X-rays, CT, lumbosacral and pelvic MRI and, in selected cases, angiography were generally performed during the early assessment of a polytraumatized patient [1, 7].

The role of evoked potentials is still debated in the literature: some authors suggest they may represent a valid adjuvant for diagnosis, monitoring (i.e. during decompression) and prediction of post-operative neurological impairment, and the panel agreed with their usefulness [2, 3, 9, 10].

Eventually, the panel agreed not to consider EMG in lower limbs in acute settings, but did agree on its use in follow-up. Even though some studies have been conducted soon after trauma, literature data support those indications indicating that EMG signs of denervation are not present immediately after the injury but they may appear later, most of the articles suggest at least 10–14 days later, because an EMG is not able to show signs of denervation in a damaged nerve until 2–4 weeks after damage [4, 10, 11, 52–54].

The panel agreed that “patient with cauda equina syndrome” (lower extremities neurological deficit, erectile dysfunction, urinary retention/urinary or faecal incontinence and saddle anesthesia) [14, 15, 44, 50] associated with a sacral fracture represents an absolute indication for sacral reduction and fixation [5, 35–38, 41].

Regarding the association between displaced sacral fractures and cauda equina syndrome, the literature hypothesizes two explanations: a displaced fracture may involve the sacral roots, causing kinking and compression, or the local haematoma may cause compression. Considering these etiologies, the literature suggests sacral laminectomy to be a possible treatment to avoid sacral roots compression, and surgical fixation following decompression should also be considered [1–3, 12]. In these cases, the surgical option should also be considered when there is failure of conservative management without recovery. On the other hand, stretching of roots, or avulsion or no evidence of canal narrowing does not represent an indication for surgery, and in these cases conservative treatment is usually recommended [13, 26]. Some authors, instead, consider cauda equina syndrome to be an absolute indication for early decompression, considering “as soon as safely possible” to be the correct timing [2]. In summary there is no consensus about cauda equina syndrome, and surgical decompression results are debated because patients with “suspected” or “incomplete” cauda equina syndrome have been enrolled, limiting the conclusions [2, 52].

There is still a lot of debate and a lack of data in the literature regarding the best approach and the correct timing for performing a sacral decompression in a patient with cauda equina syndrome. Recently, a review [3] of the clinical data did not demonstrate the benefits of surgical decompression thresholds of 24 or 48 h after symptom onset, and therefore a meaningful division with respect to timing of intervention and eventual bladder function could not be determined [7], depending on many factors including clinical presentation (e.g. saddle anesthesia is considered to be a negative predictor) and time to decompression [61, 62].

The most recurrent cut-off of 72 h has been adopted from spine surgery, some others say 48 h, and some others even say weeks; but still there are no data in support of a valid cut-off which can influence in a positive way the outcome for the patient. In this context, the panel agreed that the correct timing for reduction and fixation is “as early as possible”. The panel agreed also that the decision-making process should be influenced by the presence of a complete cauda equina syndrome or an incomplete radicular impairment [13].

In the historical literature, non-operative treatment has been the first treatment option, especially when neurological deficit was not present [7].

Surgical intervention is known to facilitate early mobilization, reduce early mortality and improve the long-term outcome [20, 27, 32, 45, 46, 51, 63]. The role of surgery in neurological recovery is still controversial [16].

Complete neurologic recovery occurs in a variable percentage of patients (46.5–62%) [25, 28, 43, 57] with abnormal immediate post-injury neurologic examination, whereas failure to recover any lost function may be seen in upward of 21.9% [15, 16].

The need for, and utility of, surgical nerve root decompression in the acute setting continues to be debated in the literature [4, 13]. Sacral roots subjected to compression, contusion, or traction caused by displacement and angulation of the sacral fracture fragments have a theoretical chance of recovery. A significant association between the presence of an incomplete neurological deficit and full recovery of neurological function has been demonstrated in the literature [13, 14]. On the other hand, no statistically significant association was noted between completeness of neurological injury and recovery of bowel and bladder function specifically (regardless of recovery of lower extremity neurological function). This discrepancy seems to be due to a propensity for complete injuries to recover bowel and bladder function without recovering extremity function [6, 16, 23].

Some authors have suggested that decompression is mandatory in the presence of canal compromise and progressive neurological deficit, regardless of the biomechanical criteria for surgery. In the presence of no progressive deficit or normal neurological status, performing surgery only for decompression has no clear benefits [2, 3, 15].

According to the panel, decompression surgery should be performed firstly through fracture reduction (indirect decompression) and laminectomy should be reserved only for worsening and progressive radicular neurological deficit of the lower limbs [2]; while in isolated and incomplete radicular neurological deficit or when there is no evidence of nerve root compression, a laminectomy after reduction is not recommended.

Regarding an early closed reduction manoeuver, only a few articles in the literature showed the results of early transcondylar traction. In selected cases a technique using manual countertraction and hyperlordosis induced by a pad positioned under the lumbo-sacral junction, has been proposed as a bridge to surgery [19, 20]. The panel agreed that a first attempt with closed reduction through external manoeuvres should not be mandatory because, in their experience, the chances of success in obtaining a good and stable reduction are low [33, 34, 45, 46].

Just a few articles are present in the literature about a possible role of transcondylar traction in displaced sacral fractures, with some authors considering this option to be a bridge to surgery. The panel agreed that it may not be useful in those patients.

We did not find evidence to support pre-operative transcondylar traction, and the panel do not suggest using it.

The literature was in favor of routinely performing fixations after surgical decompression because the decompression itself may lead to increased instability [16, 17, 25, 26].

Debate is still ongoing about which surgical fixation should be preferred; the two most common ways to perform a fixation are sacral screws vs lumbopelvic fixation. Each one has its pros and cons. For sacral screws, it is a minor invasive treatment with good stability of the implant [39, 40] and a lower risk of infections and discomfort; but this technique cannot be used in transverse sacral fractures. The pros of lumbopelvic fixation (also triangular osteosynthesis) are major stability, independent of fracture pattern, useful in insufficiency fractures with bad bone-stock in which sacral screws may not be enough. The cons include it being more expensive and a more invasive treatment with a higher risk of infection and problems with wound healing which could reach up to 16% [19, 20, 26, 48, 67].

Opinion leaders agreed that the final decision depends on the general condition of the patient, the fracture pattern and the surgeon’s preference.

The panel reached general agreement about routinely performing a stabilization after surgical decompression, but a consensus was not found about the best stabilization approach, which remains dependent on fracture pattern, patient characteristics and the surgeon’s skills.

In a patient who was not initially investigated from a neurological point of view (neither physical examination nor imaging), due to the general circumstances, but then clinical investigation after 72 h identifies a neurological deficit in the presence of a displaced sacral fracture with nerve compression on MRI, a laminectomy after reduction is indicated.

Although there is still a lot of debate and lack of data in the literature regarding delayed decompression, the general rule, as previously reported, is to perform the surgical intervention as soon as possible. Some cases have been published of patients undergoing sacral decompression more than 48–72 h after trauma in neglected neurological impairment or in failure of conservative management, and in some cases good outcomes in terms of neurological recovery have been reported [28]. To sum up, in the literature we could not find a threshold timing of surgery that conclusively correlated with outcome [15, 28]. But generally, a decompression within 48 h is considered the gold standard in the case of cauda equina syndrome [55, 56], and no improvements in outcomes have been recorded in cases where decompression was performed during the initial 24-h window in comparison to the 48-h window [29].

Neurologic symptoms associated with sacral insufficiency fractures are uncommon and occur in 2% of cases. Complete neurological deficits are exceptional. In general, neurological involvement associated with sacral insufficiency fractures resolves with the outcome of the fracture [55, 56].

As a result of the consensus, we also found that experts agreed in recommending surgical stabilization after a sacral decompression in the scenario of an insufficiency fracture, as well as in traumatic sacral fracture.

This study has several limitations; first of all, the study relies on the available literature, and the evidence base for some recommendations is limited. As noted, some statements lack consensus due to limited experience, even in highly specialized centres. Furthermore, the consensus was reached based on the opinions of experts from the Italian Pelvic Trauma Association. While expert opinions carry value, they can be influenced by individual biases and experiences, potentially affecting the reliability of the recommendations. To limit those biases, as far as possible the single surgeon background was considered and only the most skilled and experienced were included. Inter-personal influence bias was limited by individually providing the consensus questions. Some questions were not backed by robust RCTs and unfortunately relied only on case reports, retrospective studies and expert opinions, which may have inherent limitations. Moreover, the study covers a wide range of scenarios, from high-energy trauma to low-energy trauma, and includes different types of sacral fractures. The heterogeneity of the patient population and fracture patterns may impact the generalizability of the recommendations. While the consensus attempted to address various scenarios, some statements still lack agreement among the experts, indicating ongoing debates and uncertainties in the field. Further research and collection of clinical data are clearly necessary to support the best management of these patients.

## Data Availability

Data are available from the University of Turin local repository.

## Appendix

See Table 1.

**Table 1:** Statement supported by the consensus

DIAGNOSTIC PROTOCOL	
1. In case a complete neurological examination (e.g. intubated polytraumatized patient) can’t be performed, pelvic Xray are useful	
2. In case a complete neurological examination (e.g. intubated polytraumatized patient) can’t be performed, CT scan is useful	
3. In case a complete neurological examination (e.g. intubated polytraumatized patient) can’t be performed, hip and pelvis MRI is useful	
4. In case a complete neurological examination (e.g. intubated polytraumatized patient) can’t be performed, Lumbosacral MRI is useful	
5. In case a complete neurological examination (e.g. intubated polytraumatized patient) can’t be performed, Lumbosacral Evoked lower extremities potentials are useful	
6. Lower extremities EMG should be not used in an acute setting	
TREATMENT AND TIMING IN PATIENT WITH CAUDA EQUINA SYNDROME	
7. In a patient with cauda equina syndrome (lower extremities neurological deficit, erectile dysfunction, urinary retention/urinary or fecal incontinence and saddle anaesthesia) associated with a sacral fracture represents an absolute indication to sacral reduction and fixation	
8. In a patient with cauda equina syndrome (lower extremities neurological deficit, erectile dysfunction, urinary retention/urinary or fecal incontinence and saddle anaesthesia) associated with a sacral fracture, the correct timing for reduction and fixation is “as early as possible”	
TREATMENT IN PATIENT WITH HIGH ENERGY TRAUMA	
9. An isolated and complete radicular neurological deficit of the lower limbs does not represent an indication to laminectomy after reduction in case of a displaced sacral fracture in a high energy trauma associated with an imaging suggestive of root compression	
10. An isolated and incomplete radicular neurological deficit of the lower limbs does not represent an indication to laminectomy after reduction in case of a displaced sacral fracture in a high energy trauma associated with an imaging suggestive of root compression	
11. Worsening and progressive radicular neurological deficit of the lower limbs represents an indication to laminectomy after reduction in case of a displaced sacral fracture in a high energy trauma associated with an imaging suggestive of root compression	
12. In case of a displaced sacral fracture and neurological deficit with an imaging showing no evidence of nerve root compression, a laminectomy after reduction is not be indicated	
TREATMENT IN PATIENT WITH LATE DIAGNOSIS	
13. In a patient who was not initially investigated from a neurological point of view (neither physical examination nor imaging), due to the general conditions, clinical investigation after 72 hours identifies a neurological deficit in presence of a displaced sacral fracture with nerve compression on MRI, a laminectomy after reduction is indicated	
SURGICAL TECHNIQUE	
14. In case of indication to perform a sacral decompression, a first attempt with closed reduction through external manoeuvres is not mandatory	
15. Transcondylar traction does not represent a valid method of closed decompression in unstable unilateral pelvic fractures and vertical shear type fractures	
16. Following a sacral decompression, should a sacral fixation (e.g. sacroiliac screw, triangular osteosynthesis, lumbopelvic fixation) be performed	
TREATMENT IN PATIENT WITH LOW ENERGY TRAUMA	
17. An isolated and complete radicular neurological deficit of the lower limbs represents an absolute indication to laminectomy after reduction in case of a displaced sacral fracture in a low energy trauma associated with an imaging suggestive of root compression	
18. An isolated and incomplete radicular neurological deficit of the lower limbs does not represent an absolute indication to laminectomy after reduction in case of a displaced sacral fracture in a low energy trauma associated with an imaging suggestive of root compression	
19. Worsening and progressive radicular neurological deficit of the lower limbs represents an indication to laminectomy after reduction in case of a displaced sacral fracture in a low energy trauma associated with an imaging suggestive of root compression	
20. In case of a displaced sacral fracture and neurological deficit in a low energy trauma, surgical fixation after performing a sacral decompression is indicated	
